# Cooperative driving of mixed autonomy vehicles: A delay-compensating MPC approach

**DOI:** 10.1016/j.fmre.2023.12.025

**Published:** 2025-01-31

**Authors:** Xuan Wang, Yougang Bian, Mangjiang Hu, Hongmao Qin, Rongjun Ding

**Affiliations:** a The State Key Laboratory of Advanced Design and Manufacturing Technology for Vehicle, College of Mechanical and Vehicle Engineering, Hunan University, Changsha 410082, China; b Wuxi Intelligent Control Research Institute (WICRI) of Hunan University, Wuxi 214115, China

**Keywords:** Mixed autonomy, Vehicle platoon, Human-driven vehicles, Cooperative control, Model predictive control, Communication delays

## Abstract

The era of mixed autonomy when connected automated vehicles and human-driven vehicles co-exist will last a long time. Communication time delays are inevitable and may highly deteriorate the safety and stability of mixed vehicle platoons in dynamic tracking scenarios. To address this challenge, this paper proposes a model predictive control (MPC) method for cooperative control of mixed platoons with time-varying communication delays. The detriments of time delay to cooperative control are analyzed and two delay compensation mechanisms are established, based on which a dual-mode MPC controller is designed. The feasibility, closed-loop stability, and head-to-tail string stability are theoretically proved. Furthermore, numerical simulations are conducted to verify the effectiveness of the proposed MPC controller. Simulation results show that the proposed controller outperforms the state-of-the-art consensus-based controller with 4.5%-5.8% fuel consumption reduction and 28.1%-33.1% average velocity error reduction.

## Graphical abstract

**Figure:**
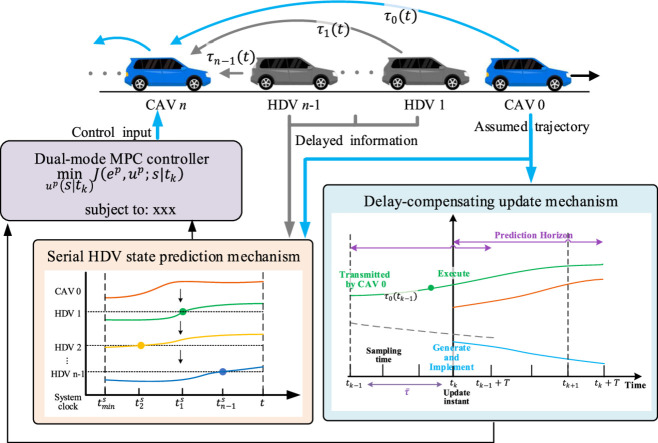

## 1 Introduction

With the rapid development of wireless communication and vehicle automation, extensive studies on the connected automated vehicle (CAV) technique have been conducted for the possibility of improving traffic flow characteristics [[1], [2], [3], [4]]. By resorting to vehicle-to-vehicle (V2 V) communication and onboard computation, vehicle platoon systems can improve safety, traffic efficiency, driving comfort, and fuel economy [5,6]. For a very long time, however, the CAV penetration rate will remain unsaturated, leading to the coexistence of CAVs and human-driven vehicles (HDVs) on the road [[7], [8], [9]]. Hence, the cooperative control of mixed vehicle platoons deserves further research.

In mixed platoons, recurring acceleration and deceleration of HDVs will lead to traffic oscillations, also known as “stop-and-go”, which may cause road congestion [10]. The collective dynamics of HDVs can induce traffic jams without any external disturbances such as lane changes and intersections [11]. Recently, theoretical and experimental results have shown that traffic waves can be dissipated by incorporating CAVs with a low penetration rate into traffic flow in intelligent connected road environment [12]. Wang et al. first analyzed the controllability, stabilizability, and reachability of a mixed traffic system, which is fundamental for mixed platoon control [13]. Wang et al. built a nonlinear mixed traffic model and proposed an optimal controller with the objective of minimizing speed perturbation to smooth unstable traffic flow [14]. Since HDVs cannot be controlled directly and do not follow deterministic behavior, unknown HDV models have recently received significant attention. Ding et al. trained a sparrow search algorithm-based Elman neural network with a large amount of real-world car-following data to estimate multiple HDVs’ states and design feedback following strategy for CAVs [15]. Wang et al. built a data-centric mixed traffic model utilizing the Willems’ fundamental lemma and proposed an optimal controller with a receding horizon strategy, of which the performance was validated with numerical experiments and nonlinear traffic simulations [16,17]. Numerous efforts have been made to study robustness against environmental uncertainties, prediction uncertainty of HDVs and noisy data. He et al. proposed a constrained adversarial reinforcement learning approach for decision making of CAVs at highway on-ramps with ensuring robustness against environmental uncertainties [18]. Feng et al. denoted the prediction uncertainty of the preceding HDV as an unknown tracking error and proposed a tube model predictive controller (TMPC) to control following CAVs robustly and efficiently [19]. In this case, HDVs are not connected and only the preceding HDV can be measured by onboard sensors. Lan et al. constructed an over-approximation model with noisy platoon data for a mixed platoon system and adopted the data-driven reachability technique to predict future state, based on which a robust model predictive control (RMPC) was designed with provably guaranteeing the safe and robust performance [20].

V2 V inevitably introduces communication time delays in vehicle platoons due to electronic noise, network congestion, and complex traffic conditions such as tunnels and architectural barriers [21]. Communication time delays may vary as a result of processing, queuing, transmission, and random package loss [22]. This phenomenon may inhibit platoon performance and even render closed-loop stability and string stability invalid [23]. Fiengo et al. proposed a strategy by explicitly considering time-varying communication delays for deducing traffic waves, and asymptotic stability and head-to-tail stability were strictly proved [24]. Di et al. further presented the tuning procedure of the control actions and VANET simulator-based validation [25]. Ge et al. adopted linear quadratic regulation (LQR) to obtain an optimal feedback controller for mixed platoon systems with communication time delays and driver reaction time delays, and head-to-tail stability can be guaranteed with appropriate weighting factors [26]. Ge et al. further estimated HDV parameters using experimental data with a sweeping least square method [27]. Guo et al. proposed an inverse MPC-based anticipation approach to predict HDVs’ state and integrated it into an MPC controller for CAV control in communication-constrained traffic, whereas in this case, only one HDV was involved in the platoon [28].

It should be noticed that existing delay compensation strategies often resort to prediction [21,28,29,30]. Furthermore, MPC has an innate feature to compensate for communication time delay because of the optimal control strategy in a finite horizon [31,32]. To the best of the authors’ knowledge, there is a lack of delay compensation in mixed platoon control under the MPC architecture. Therefore, this paper focuses on delay compensation and MPC controller design for mixed platoons. Compared to the feedback controller [24,25], safety constraints and optimal performance can be explicitly guaranteed and theoretically proved [[33], [34], [35]].

In this study, an MPC-based control scheme with robustness against time-varying heterogenous communication delays and external input for mixed platoons is developed. Specifically, third-order heterogeneous car-following dynamics of CAVs and HDVs are integrated into the system model to improve control accuracy. The dynamic behavior of the leader vehicle is explicitly modeled as a time-varying and known disturbance to improve tracking performance. Two delay compensation mechanisms are established to eliminate the detriments of communication delays, based on which a dual-mode MPC controller is designed. Feasibility, closed-loop stability, and head-to-tail string stability are theoretically guaranteed by proper constraint design and parameter selection of control invariant set. Concerning the existing literature, the main contributions of this work are summarized as follows.

(1) To eliminate the detriments of communication delays, the challenges brought by heterogeneous communication delays to HDV prediction and MPC controller design are explicitly analyzed, then a serial HDV state prediction mechanism and a delay-compensating update mechanism are established to address the challenges.
(2) By incorporating the serial HDV state prediction mechanism and delay-compensating update mechanism, a dual-mode MPC controller is proposed with robustness against communication delays for mixed platoons. The feasibility, closed-loop stability, and head-to-tail string stability are theoretically guaranteed. Compared to the consensus approach without delay compensation [24,25], the proposed approach has better performance in terms of safety, fuel economy, and dynamic tracking.
(3) The dynamic behavior of the leading vehicle will drift the mixed platoon system away from the equilibrium state. To enhance robustness against external input, the proposed control scheme explicitly considers a dynamic CAV leader, of which the predictive trajectory can be shared within the mixed platoon. Compared to [17] which only provides stability guarantee at the equilibrium, the proposed method is more practical for dynamic tracking scenarios in real mixed traffic.

The rest of the paper is organized as follows: Section 2 introduces the problem formulation of mixed platoon control and preliminaries, where the main results are given in Section 3. Section 4 presents feasibility analysis and Section 5 gives stability analysis of the closed-loop system. Simulation results are provided in Section 6, and conclusions are drawn in Section 7.

## 2 Problem formation and preliminaries

Notations: Denote by N, R, and N the fields of integers, real numbers, and the collection of integers 1,…,N, respectively. $R^{n}$ is the n-dimensional Euclidean space and ⊗ is the Kronecker product. Define ∥x∥QΔ=xTQx, where $x\in R^{n}$ and $Q\in R^{n\times n}$ is a positive definite matrix. Denote by x(k|t) the value of x(t+k) predicted at time t.

### 2.1 Problem formation

**Fig. 1:**
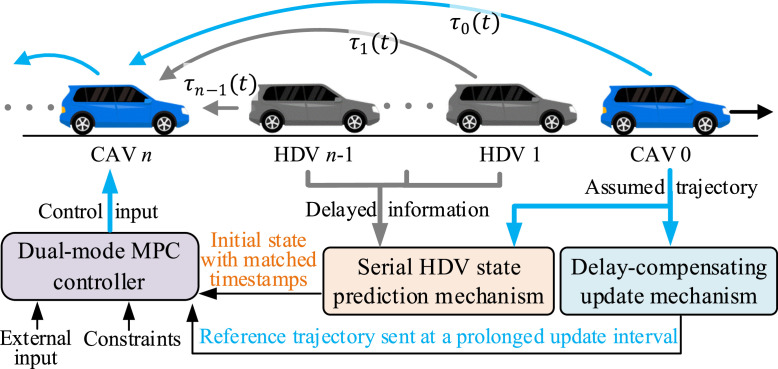
**A mixed platoon under time delays with the proposed control scheme**.

As shown in Fig. 1, we consider a mixed platoon running on a straight, flat road with a leading CAV 0, n−1 HDVs and a following CAV n [19,20]. All CAVs and HDVs are equipped with V2 V communication devices. CAV 0 may be the tail vehicle in a platoon or following a series of HDVs. The scenario studied in this paper is general since a long-mixed traffic flow can be split into multiple platoons or sub-systems with the following formation. CAV 0 can ensure controllability and provide a reference trajectory for HDV prediction. CAV 0 may be forced to adjust its trajectory by intersection, cut-in, and cut-out in the downstream traffic flow, resulting in external input to the mixed platoon system. With dynamic external input and heterogeneous communication delays, how to control CAV n robustly and efficiently remains a huge challenge, which is the focus of this paper.

The dynamics of CAV i is

$${\dot{p}}_{i}=v_{i},$$

$${\dot{v}}_{i}=a_{i},$$

(1) $${\dot{a}}_{i}=\frac{1}{\delta_{i}}\left(u_{i}-a_{i}\right),\;i\in \left\{0\right\}\cup \left\{n\right\},$$

where $p_{i},\;v_{i},\delta_{i},\;a_{i},$ and $u_{i}$ are the position, velocity, constant actuation time lag, acceleration, and control input, respectively.

The third-order nonlinear car-following model is used to capture HDVs’ behavior:

$${\dot{h}}_{i}=v_{i-1}-v_{i},$$

$${\dot{v}}_{i}=a_{i},$$

(2) $${\dot{a}}_{i}=\frac{1}{\delta_{i}}\left(\alpha_{i}\left(g\left(h_{i}\right)-v_{i}\right)+\beta_{i}\left(v_{i-1}-v_{i}\right)-a_{i}\right)$$

where $h_{i}=p_{i-1}-p_{i}$ is the relative spacing between HDV i and its predecessor i−1, $\alpha_{i}$ and $\beta_{i}$ are response gains to the relative spacing and relative velocity. $g(h_{i})$ is a smooth sigmoid function to map the spacing into desired velocity given by [36]

$$g\left(h_{i}\right)=\left\{ \begin{array}{cc} 0, & h_{i}\leq h_{s}\\ \frac{v_{max}}{2}\left[1-\text{cos}\left(\pi \frac{h_{i}-h_{s}}{h_{g}-h_{s}}\right)\right], & h_{s}<h_{i}<h_{g}\\ v_{max}, & h_{i}\geq h_{g} \end{array}\right.,$$

where $h_{s}$,$\;h_{g}$, and $v_{max}$ are the smallest spacing before stopping, the largest spacing, and the maximum velocity, respectively.

The HDVs will ultimately reach the desired equilibrium state ($h^{*},v^{*}$) following a constant-velocity leading CAV, where the desired velocity $v^{*}=v_{0}$ and the desired spacing $h^{*}$ is obtained by $v^{*}=g\left(h^{*}\right)$. By linearizing Eq. 2 at equilibrium state ($h^{*},v^{*}$) and defining the system state as $x_{i}={\left[\Delta h_{i},\Delta v_{i},a_{i}\right]}^{T}$, we can obtain the following model of HDVs:

(3) $${\dot{x}}_{i}=A_{i}x_{i}+D_{i}x_{i-1},\;i\in \left\{1,\ldots ,n-1\right\},$$

where $\Delta h_{i}=h_{i}-h^{*},\Delta v_{i}=v_{i}-v^{*},$

$$A_{i}=\left[\begin{array}{ccc} 0 & -1 & 0\\ 0 & 0 & 1\\ \frac{{\bar{a}}_{i}}{\delta_{i}} & \frac{-{\bar{\beta }}_{i}}{\delta_{i}} & \frac{-1}{\delta_{i}} \end{array}\right],\;D_{1}=0,\;D_{i}=\left[\begin{array}{ccc} 0 & 1 & 0\\ 0 & 0 & 0\\ 0 & \frac{{\bar{c}}_{i}}{\delta_{i}} & 0 \end{array}\right],$$

${\bar{\alpha }}_{i}=\alpha_{i}g^{\prime }\left(h^{*}\right),{\bar{\beta }}_{i}=\alpha_{i}+\beta_{i},{\bar{c}}_{i}=\beta_{i},$ and $g^{\prime }\left(h^{*}\right)$ denotes the derivative of $g(h_{i})$ with respect to $h_{i}$ at $h^{*}$.

Then we can obtain the linear overall state-space model for the mixed platoon system as

(4) $$\dot{x}=Ax+Bu+Ca_{0},$$

with the coefficient matrices

$$A=\left[\begin{array}{cccc} A_{1} & & & \\ D_{2} & A_{2} & & \\ & \ddots & \ddots & \\ & & D_{n} & A_{n} \end{array}\right],B=\left[\begin{array}{c} B_{1}\\ B_{2}\\ \vdots \\ B_{n} \end{array}\right],\;C=\left[\begin{array}{c} C_{1}\\ C_{2}\\ \vdots \\ C_{n} \end{array}\right],$$

$$B_{i}=0,C_{i}=0,\;i\in \left\{1,\ldots ,n-1\right\},$$

$$A_{n}=\left[\begin{array}{ccc} 0 & -1 & 0\\ 0 & 0 & 1\\ 0 & 0 & -\frac{1}{\delta_{n}} \end{array}\right],B_{n}=\left[\begin{array}{c} 0\\ 0\\ \frac{1}{\delta_{n}} \end{array}\right],\;D_{n}=\left[\begin{array}{ccc} 0 & 1 & 0\\ 0 & 0 & 0\\ 0 & 0 & 0 \end{array}\right],C_{n}=\left[\begin{array}{c} 0\\ -1\\ 0 \end{array}\right],$$

where $x={\left[x_{1}^{T}\ldots \;x_{n}^{T}\right]}^{T}$ is the overall state vector and $u=u_{n}$ is the control input to be designed.

Then define the overall tracking error as $e={\left[e_{1}^{T}\ldots \;e_{n}^{T}\right]}^{T}$ with $e_{i}=x_{i}-{\left[0,0,a_{0}\right]}^{T}$. Based on Eq. 1 and Eq. 4, we can obtain

(5) $$\dot{e}=Ae+Bu+d_{0},$$

where $d_{0}=Ea_{0}+Fu_{0}$ with the coefficient matrices

$$\begin{array}{ccc} E & = & \left[\begin{array}{c} E_{1}\\ E_{2}\\ \vdots \\ E_{n} \end{array}\right],\;F=\left[\begin{array}{c} F_{1}\\ F_{2}\\ \vdots \\ F_{n} \end{array}\right],\;E_{i}=\left[\begin{array}{c} 0\\ 0\\ \frac{1}{\delta_{0}} \end{array}\right],\;F_{i}=\left[\begin{array}{c} 0\\ 0\\ -\frac{1}{\delta_{0}} \end{array}\right],\\ i & \in & \left\{1,\ldots ,n-1\right\},\;E_{n}=\left[\begin{array}{c} 0\\ -1\\ \frac{1}{\delta_{0}} \end{array}\right],F_{n}=\left[\begin{array}{c} 0\\ 0\\ -\frac{1}{\delta_{0}} \end{array}\right]. \end{array}$$

State-space model Eq. 5 has been widely applied in platoon control of full CAVs and mixed autonomy with practical validation [16,37,38]. By proper design of MPC controller, excellent control performance and low computational burden can be achieved simultaneously [38].

Remark 1 External input $d_{0}$ is the combination of control input and acceleration of CAV 0, which will drift the system away from the equilibrium state. Most existing works in mixed platoons assume that $d_{0}$ is zero or unknown disturbance, [17,20]. In the proposed control scheme, $d_{0}$ can be transmitted by V2 V communication of downstream CAV (see Section 3) or pre-planned by ecologic speed planning methodology [39] if there is no downstream CAV. Therefore, it can be regarded as a known disturbance into the mixed platoon system.

Remark 2 It's noted that compared to the system model based on the second-order optimal velocity model (OVM) [25], Eq. 5 includes heterogeneous actuation time lag to build a more realistic mixed platoon model.

### 2.2 Control objective

The control objective in this study is to design an MPC-based controller for mixed platoon system Eq. 4 to be robust against heterogeneous communication time delays and to meet the following criteria.

(1) Closed-loop stability: The tracking error of mixed platoon system is within a specified bound under time-varying external input $d_{0}$, i.e., $\lim_{t\to +\infty }e\left(t\right)\in \Omega \left(\varepsilon \right)$.

where Ω(ε) is a control invariant set and its size depends on the external input and controller parameters (see Section 2.3).

(2) Head-to-tail string stability: The velocity fluctuations are suppressed from the head CAV to the tail CAV with a bound, i.e., $|\Delta v_{n}|\leq \Delta v_{max}$, where $\Delta v_{max}$ is the maximum allowable velocity error. It is worth noted that this definition is in the sense of $L_{2}$ string stability [20].
(3) Driving Safety: Satisfy tracking error constraint is satisfied:
  (6) e(t)∈E,
  where $E=\{e\in R^{3n}|I_{n}⊗e_{min}\leq e\leq I_{n}⊗e_{max}\}$ with $e_{min}={\left[\Delta h_{min},\Delta v_{min},\Delta a_{min}\right]}^{T}$ and $e_{max}={\left[\Delta h_{max},\Delta v_{max},\Delta a_{max}\right]}^{T}$. $\Delta h_{min},\Delta v_{min},\Delta a_{min}$ represent the lower bounds of spacing error, velocity error, and acceleration error, respectively, and $\Delta h_{max},\Delta v_{max},\Delta a_{max}$ represent the upper bounds of spacing error, velocity error, and acceleration error, respectively. Obviously, the tracking error constraint Eq. 6 can explicitly avoid collisions by selecting a safe $\Delta h_{min}$. It is noted that the constraints only take effect on CAV n since HDVs are controlled by human drivers.

### 2.3 Preliminaries

Assumption 1 The communication delays in the mixed platoon system are time-varying and heterogeneous but bounded (see Fig. 1).

Definition 1 (control invariant set [40,37]): A set C⊂E is called a control invariant set for system Eq. 5 subject to constraint Eq. 6 if

$$\begin{array}{c} e\left(t\right)\in E\Rightarrow \exists u\left(t\right)\in U\\ \text{subj}.\;\text{to}\;Ax\left(t\right)+Bu\left(t\right)+d_{0}\left(t\right)\in C,\\ \forall d_{0}\left(t\right)\in D,\forall t\geq 0, \end{array}$$

where $D=\{d_{0}\in R∥d_{0}|\leq d_{max}\}$ and $d_{max}$ is the upper bound of external input $d_{0}$. Further, the set $C_{\infty }$ is said to be the maximal control invariant set if it contains all control invariant set contained in E.

Lemma 1 *For system (*5*), there exist constant* ε>0*, matrices* Q>0,R>0,P>0*, and a state feedback gain* K *such that the following hold: 1) the set* Ω(ε)Δ={e(t)|V(e(t))≤ε2}*, is a control invariant set for the system* $\dot{e}\left(t\right)=A^{c}e\left(t\right)$*; 2) for any* e(t)∈Ω(ε)*, the inequality* $\dot{V}\left(e(t)\right)\leq -{∥e\left(t\right)∥}_{Q^{*}}^{2}$ *holds. where* $V\left(e(t)\right)={∥e\left(t\right)∥}_{P}^{2}$*,* $Q^{*}=Q+K^{T}RT$ *and* $A^{c}=A+BK$.

Remark 3 The linear quadratic regulator (LQR) can be applied to select K. The set Ω(ε) can be designed as a maximal positive control invariant set and the calculation steps can be found in [37].

### 2.4 Challenges

The existence of heterogeneous communication delays brings two challenges:

(1) Challenge to HDV state prediction: In most existing studies, the state prediction of n HDVs is based on the aggregated state-space model of a series of HDVs. Heterogeneous communication delays will lead to timestamps mismatch between the information transmitted by HDVs, which may reduce prediction accuracy and produce unsatisfactory control effects.
(2) Challenge to MPC-based controller design: In general, the control signals are generated at each sampling time, so all required information is assumed to be received at each sampling time explicitly or implicitly. However, it cannot be established when communication delays exist. More specifically, when time-varying communication delays are greater than the sampling interval, the information sent by CAV 0 and HDVs at time k cannot be received by CAV n at k+1, where k and k+1 are adjacent sampling times. In this case, the past information will be applied to generate control signals, which may deteriorate control performance and even affect safety.

## 3 Main results

This section presents the main results. First, we present a serial HDV state prediction mechanism to address the challenge of HDV prediction. Next, a delay-compensating update mechanism is established to address the challenge of MPC-based controller design, based on which a dual-mode MPC controller is proposed.

### 3.1 Serial HDV state prediction mechanism

**Fig. 2:**
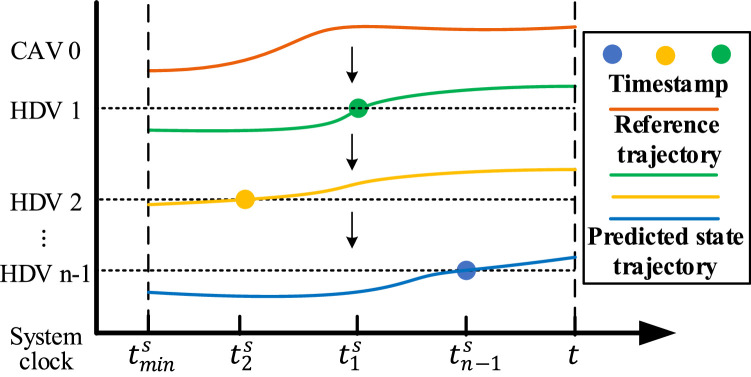
**The serial HDV state prediction mechanism**.

As shown in Fig. 2, the timestamps $t_{k}^{s},\;k=1,2,\ldots ,n-1$ of the latest HDVs’ data are mismatched due to heterogeneous communication delays. At time t, the initial state e(t) is required for controller design, so we need to predict the HDV state to match the timestamps between HDVs and CAV n’s measurement. Denote $t_{min}^{s}$ as the smallest timestamps of all HDVs’ data at time t. According to HDV model (3), the state trajectory of HDV 1 in $\left(t_{min}^{s},t\right)$ can be generated based on the reference trajectory of CAV 0 and the latest data $x_{1}\left(t_{1}^{s}\right)$, then subsequent HDVs state trajectory can be generated based on that of the predecessor one by one until HDV n−1. Finally, we can obtain the initial state e(t) for controller design.

Remark 4 The serial HDV state prediction mechanism is irrelevant to the HDV model. Therefore, some existing work based on Intelligent Driver Model (IDM) [41], Artificial Neural Networks (ANN) [42], and Inverse Model Predictive Control (IMPC) [28] may also incorporate this mechanism to address timestamps mismatch in HDV prediction and improve prediction accuracy under heterogeneous communication delays.

Remark 5 The prediction error of HDVs is inevitable, which is handled implicitly by rolling optimization of MPC in this work. Research on handling the prediction uncertainty of HDVs explicitly can be found in [19].

### 3.2 Delay-compensating update mechanism

**Fig. 3:**
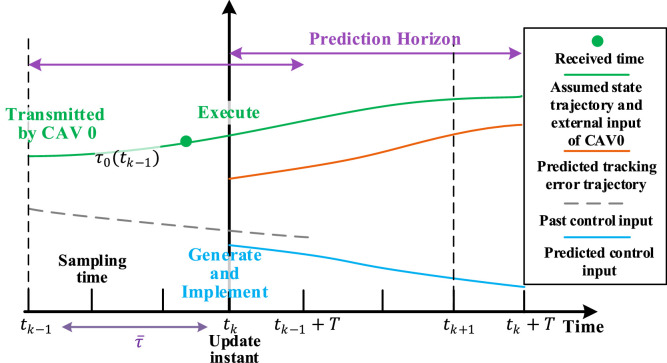
**The delay-compensating update mechanism**.

We divide the time domain by the sampling time k,k=0,1,2,… with sampling interval θ>0. To eliminate the negative effect of communication time delay on receding horizon optimization, a delay-compensating update mechanism is designed as below. According to Assumption 1, the heterogeneous communication delays are time-varying but bounded, i.e., $\tau_{i}\left(t\right)\leq \bar{\tau }=n\cdot \theta ,\;n\in N$, then define $t_{k},k=0,1,\ldots$ as the update instants when CAV n solve an optimization problem, generate the control signals and send the assumed state trajectory and external input to upstream CAV. To guarantee that CAV n can receive the information from CAV 0 at update instants under communication delays, we set the update interval as delay upper bound, i.e., $t_{k+1}-t_{k}=\bar{\tau }$. By solving the optimization problem with all required information, CAV n implements the optimal control input sequence generated at time $t_{k}$, and re-computes the optimal control input at next update instant $t_{k+1}$. The delay-compensating update mechanism is illustrated in Fig. 3.

Remark 6 In general, the control signals are generated at each sampling time and only the first control input is implemented. To eliminate the influence of time-varying communication delay on receding optimization, the interval for generating control signals (update interval) is prolonged as the delay upper bound, and a sequence of control input is implemented during update intervals. Therefore, the feasibility and stability analysis of MPC with communication delays become rather challenging.

### 3.3 Dual-mode MPC controller design

The serial HDV state prediction mechanism and delay-compensating update mechanism address the timestamps mismatch and provide the latest information of CAV 0 and HDVs. Then at time $t_{k}$, the optimal control input trajectory can be generated by solving the following optimization problem P.

The serial HDV state prediction mechanism and delay-compensating update mechanism address the timestamps mismatch and provide the latest information of CAV 0 and HDVs. Then at time $t_{k}$, the optimal control input trajectory can be generated by solving the following optimization problem P

(7a) $$\begin{array}{ccc} & & \underset{u^{p}\left(s|t_{k}\right)}{\text{min}}J\left(e^{p},u^{p};s|t_{k}\right)\\ & & =\int_{t_{k}}^{t_{k}+T}\left(∥e^{p}\left(s|t_{k}\right){∥}_{Q}^{2}+{∥u^{p}\left(s|t_{k}\right)∥}_{R}^{2}\right)ds+{∥e^{p}\left(t_{k}+T|t_{k}\right)∥}_{P}^{2}, \end{array}$$

subject to:

(7b) $$e^{p}\left(t_{k}|t_{k}\right)=e\left(t_{k}\right),$$

(7c) $${\dot{e}}^{p}\left(s|t_{k}\right)=Ae^{p}\left(s|t_{k}\right)+Bu^{p}\left(s|t_{k}\right)+d_{0}\left(s|t_{k}\right),$$

(7d) $$d_{0}\left(s|t_{k}\right)=Ea_{0}\left(s|t_{k}\right)+Fu_{0}\left(s|t_{k}\right)\in D,$$

(7e) $$u^{p}\left(s|t_{k}\right)\in U,$$

(7f) $$e^{p}\left(s|t_{k}\right)\in E,$$

(7g) $$∥e^{p}\left(s|t_{k}\right){∥}_{P}\leq \frac{\gamma T}{s-t_{k}}\varepsilon ,\;s\in \left(t_{k},t_{k}+T\right],$$

where $e(t_{k})$ is generated by the serial HDV state prediction mechanism, $e^{p}\left(s|t_{k}\right),\;u^{p}\left(s|t_{k}\right)$ are predicted tracking error trajectory and predicted control input trajectory. The matrices Q,R,P are determined by Lemma 1. 0<γ<1 is a constant parameter. It is noted that $e(t_{k})$ is calculated with the measurement of CAV n, information from CAV 0, and the prediction of HDVs by serial HDV state prediction mechanism.

The cost function consists of three items: 1) the penalty on the tracking error with a positive weight matrix Q; 2) the penalty on the predicted control input with a positive weight matrix R; 3) the terminal penalty on the deviation between the predicted and desired states with a positive weight matrix P.

Constraints Eq. 7b and Eq. 7c are the initial tracking error constraint and system dynamics constraint, respectively. Constraints Eq. 7d is the future trajectory of external input $d_{0}$, which can be obtained by V2 V communication. Constraint Eq. 7e is the control input constraint, where $U=\{u\in R|u_{min}\leq u\leq u_{max}\}$, $u_{min}$ and $u_{max}$ represent the lower bound and upper bound of control input. Constraint Eq. 7f is tracking error constraint given in Eq. 6. Constraint Eq. 7g) is the robustness constraint to enhance the closed-loop stability [43], which indicates that the tracking error will not exceed a given bound and will enter the terminal set at each prediction horizon.

For CAV *n* at time $t_{k}$, generate the assumed tracking error trajectory at $t_{k+1}$ by

$${\dot{e}}^{a}\left(s|t_{k+1}\right)=Ae^{a}\left(s|t_{k+1}\right)+Bu^{a}\left(s|t_{k+1}\right)+d_{0}\left(s|t_{k+1}\right),$$

$$s\in \left[t_{k+1},t_{k+1}+T\right],$$

$$e^{a}\left(t_{k+1}|t_{k+1}\right)=e^{*}\left(t_{k+1}|t_{k}\right),$$

$$d_{0}\left(s|t_{k+1}\right)=Ea_{0}\left(s|t_{k}\right)+Fu_{0}\left(s|t_{k}\right)\in D,$$

$$s\in \left(t_{k+1},t_{k}+T\right],$$

(8) $$d_{0}\left(s|t_{k+1}\right)=0\in D,\;s\in \left(t_{k}+T,t_{k+1}+T\right],$$

where the assumed input trajectory is generated as follows according to [43] [44]:

(9) $$u^{a}\left(s|t_{k+1}\right)=\left\{ \begin{array}{c} u^{*}\left(s|t_{k}\right),\;s\in \left[t_{k+1},t_{k}+T\right],\\ Ke^{a}\left(s|t_{k+1}\right),\;s\in \left(t_{k}+T,t_{k+1}+T\right], \end{array}\right.$$

and K is determined by Lemma 1. It should be noted that for $s\in (t_{k}+T,t_{k+1}+T]$, $u^{a}$ and $e^{a}$ can be generated alternatively by Eq. 8 and Eq. 9. Then the assumed acceleration of CAV n at $t_{k+1}$ can be generated by Eq. 9 and dynamics Eq. 1, after which $d_{n}\left(s|t_{k+1}\right)$ can be obtained and transmitted to upstream CAV in the following mixed platoon system.

Remark 7 Most existing optimal control-based works in mixed platoons assume that the external input caused by the leader vehicle is zero or unknown disturbance [17,20] which may lead to inaccuracy or conservatism. In this study, the external input $d_{0}$ is time-varying and known in each prediction horizon by making full use of prediction and V2 V communication. With explicit consideration in the prediction model Eq. 7c, the feasibility and control objective in Section 2.2 are achieved. This may enable faster convergence in dynamic leader tracking scenarios.

Remark 8 At each control instant, $d_{n}\left(s|t_{k+1}\right)$ can be generated and transmitted to the upstream CAV, which ensures that optimization problem P can be completed for all tail CAVs in the mixed platoon system.

## 4 Feasibility analysis

To prove the recursive feasibility of the proposed MPC scheme, the following assumption is made.

Assumption 2 For each CAV n, given the prediction horizon $T>\bar{\tau }$, there exists a feasible solution to optimization problem P for some initial state $e^{0}\in Z_{i}$, where $Z_{i}$ is the feasible set including all the initial states such that Problem P admits a feasible solution [43,44].

Theorem 1 *Suppose that* Assumptions 1*–*2 *hold for system (*5*), then the proposed MPC is recursively feasible for all* $t_{k},\;k\geq 1$.

Proof Given Assumption 2, we know that at time $t_{k},\;k\geq 1$ for CAV *n*, the optimal solution $u^{*}\left(s|t_{k}\right)$ of the optimization problem P exits. Then we only need to prove that there always exists a feasible solution to P at time $t_{k+1}$ based on $u^{*}\left(s|t_{k}\right)$. We choose the feasible solution candidate at time $t_{k+1}$ as [43,45]

(10) $$\tilde{u}\left(s|t_{k+1}\right)=\left\{ \begin{array}{c} u^{*}\left(s|t_{k}\right),\;s\in \left[t_{k+1},t_{k}+T\right],\\ K\tilde{e}\left(s|t_{k+1}\right),s\in \left(t_{k}+T,t_{k+1}+T\right], \end{array}\right.$$

where $\tilde{e}\left(s|t_{k+1}\right)$ is the tracking error trajectory generated by the feasible input trajectory candidate $\tilde{u}\left(s|t_{k+1}\right)$ as

$$\dot{\tilde{e}}\left(s|t_{k+1}\right)=A\tilde{e}\left(s|t_{k+1}\right)+B\tilde{e}\left(s|t_{k+1}\right)+d_{0}\left(s|t_{k+1}\right),$$

$$s\in \left[t_{k+1},t_{k+1}+T\right],$$

$$\tilde{e}\left(t_{k+1}|t_{k+1}\right)=e^{*}\left(t_{k+1}|t_{k}\right),$$

$$d_{0}\left(s|t_{k+1}\right)=Ea_{0}\left(s|t_{k}\right)+Fu_{0}\left(s|t_{k}\right)\in D,$$

$$s\in \left(t_{k+1},t_{k}+T\right],$$

(11) $$d_{0}\left(s|t_{k+1}\right)=0\in D,\;s\in \left(t_{k}+T,t_{k+1}+T\right],$$

It should be noted that for $s\in (t_{k}+T,\;t_{k+1}+T]$, $\tilde{u}\left(s|t_{k+1}\right)$ and $\tilde{e}\left(s|t_{k+1}\right)$ can be generated alternatively similar to Eq. 8 and Eq. 9. Hereinafter, we will prove that $\tilde{u}\left(s|t_{k+1}\right),\;s\in \left[t_{k+1},\;t_{k+1}+T\right]$ is indeed a feasible solution at time $t_{k+1}$.

1) The control input constraint Eq. 7e is satisfied.

For $s_{1}\in \left[t_{k+1},\;t_{k}+T\right]$, since $u^{*}\left(s_{1}|t_{k}\right)$ is the optimal solution to P at time $t_{k}$, we have $\tilde{u}\left(s_{1}|t_{k+1}\right)\in U$; For $s_{2}\in \left(t_{k}+T,\;t_{k+1}+T\right]$, generated by the feasible input trajectory candidate, $\tilde{e}\left(t_{k}+T|t_{k+1}\right)=e^{*}\left(t_{k}+T|t_{k}\right)$, where $e^{*}\left(s|t_{k}\right)$ is the optimal state trajectory of P at time $t_{k}$. According to the robustness constraint Eq. 7g, we have $∥e^{*}\left(t_{k}+T|t_{k}\right){∥}_{P}\leq \alpha \varepsilon$, so $V\left(e^{*}\left(t_{k}+T|t_{k}\right)\right)\leq \varepsilon^{2}$, which means $e^{*}\left(t_{k}+T|t_{k}\right)\in \Omega \left(\varepsilon \right)$. Then according to Lemma 1, we have $\tilde{u}\left(s_{2}|t_{k+1}\right)\in U_{i}$.

2) The tracking error constraint Eq. 7f is satisfied.

For $s_{1}\in \left[t_{k+1},\;t_{k}+T\right]$, we have $\tilde{e}\left(s_{1}|t_{k+1}\right)=e^{*}\left(s_{1}|t_{k}\right)\in E$; For $s_{2}\in \left(t_{k}+T,\;t_{k+1}+T\right]$, we have $\tilde{e}\left(t_{k}+T|t_{k+1}\right)=e^{*}\left(t_{k}+T|t_{k}\right)\in \Omega \left(\varepsilon \right)$, since $d_{0}\left(s_{2}|t_{k+1}\right)=0\in D$, according to Definition 1 and Lemma 1, we have $\tilde{e}\left(s_{2}|t_{k+1}\right)\in E$.

3) The robustness constraint Eq. 7g is satisfied.

For $s_{1}\in \left[t_{k+1},\;t_{k}+T\right]$, we have $∥\tilde{e}\left(s_{1}|t_{k+1}\right){∥}_{P}=∥e^{*}\left(s_{1}|t_{k}\right){∥}_{P}\leq \frac{T\alpha }{s_{1}-t_{k}}\varepsilon \leq \frac{T\alpha }{s_{1}-t_{k}+1}\varepsilon$;

For $s_{2}\in \left(t_{k}+T,\;t_{k+1}+T\right]$, the feedback control law $K\tilde{e}\left(s_{2}|t_{k+1}\right)$ is applied, then according to Lemma 1 and the comparison principle, the upper bound of the feasible state candidate $\tilde{e}\left(s_{2}|t_{k+1}\right)$ can be derived as [46]

$$∥\tilde{e}\left(s_{2}|t_{k+1}\right){∥}_{P}\leq F\left(s_{2}\right),\;s_{2}\in \left(t_{k}+T,\;t_{k+1}+T\right],$$

where F(s2)Δ=αεe−λ‾(Q*)(s2−tk−T)/(2λ¯(P)). For $s_{2}\in \left(t_{k}+T,\;t_{k+1}+T\right]$, we have $∥\tilde{e}\left(s_{2}|t_{k+1}\right){∥}_{P}\leq F\left(s_{2}\right)\leq \frac{T\alpha }{s_{2}-t_{k}+1}\varepsilon$.

To sum up, we can obtain that the input constraint, the tracking error constraint, and the robustness constraint are all satisfied at time $t_{k+1}$, which indicates that $\tilde{u}\left(s|t_{k+1}\right),\;s\in \left[t_{k+1},\;t_{k+1}+T\right]$ is a feasible solution to the optimization problem P. Then we finish the proof.  □

## 5 Stability analysis

This section presents the stability analysis for the closed-loop system. Two theorems are proposed for closed-loop stability and head-to-tail string stability respectively.

Theorem 2 *Suppose* Assumptions 1*–*2 *hold, then system (*5*) with the proposed MPC controller (7) achieves closed-loop stability*.

Proof The proof is divided into two sections according to the two modes of MPC, i.e., outside the terminal set and in the terminal set.

1) $e^{*}\left(s|t_{k}\right)\in Z\setminus \Omega \left(\varepsilon \right)$

Denote the optimal cost function at time $t_{k}$ by

$$\begin{array}{ccc} J^{*}\left(t_{k}\right) & = & J\left(e^{*},u^{*};s|t_{k}\right)\\ & = & \int_{t_{k}}^{t_{k}+T}\left(∥e^{*}\left(s|t_{k}\right){∥}_{Q}^{2}+{∥u^{*}\left(s|t_{k}\right)∥}_{R}^{2}\right)ds+{∥e^{*}\left(t_{k}+T|t_{k}\right)∥}_{P}^{2}, \end{array}$$

and the increment of $J^{*}\left(t_{k}\right)$ by

$$\Delta J^{*}\left(t_{k}\right)=J^{*}\left(t_{k+1}\right)-J^{*}\left(t_{k}\right)\leq \tilde{J}\left(t_{k+1}\right)-J^{*}\left(t_{k}\right),$$

where $\tilde{J}\left(t_{k+1}\right)=J\left(\tilde{e},\tilde{u};s|t_{k}\right)$ is the cost function using the candidate control input, and the inequality applies to the optimality of $J^{*}\left(t_{k+1}\right)$. Then we have

$$\begin{array}{ccc} & & \Delta J^{*}\left(t_{k}\right)\leq \int_{t_{k}+T}^{t_{k+1}+T}∥\tilde{e}\left(s|t_{k+1}\right){∥}_{Q}^{2}+∥\tilde{u}\left(s|t_{k+1}\right){∥}_{R}^{2}ds+\int_{t_{k+1}}^{t_{k}+T}{∥\tilde{e}\left(s|t_{k+1}\right)∥}_{Q}^{2}\\ & & +\;∥\tilde{u}\left(s|t_{k+1}\right){∥}_{R}^{2}ds-\int_{t_{k+1}}^{t_{k}+T}∥e^{*}\left(s|t_{k}\right){∥}_{Q}^{2}+{∥u^{*}\left(s|t_{k}\right)∥}_{R}^{2}ds\\ & & -\int_{t_{k}}^{t_{k+1}}\;\;∥e^{*}\left(s|t_{k}\right){∥}_{Q}^{2}+∥u^{*}\left(s|t_{k}\right){∥}_{R}^{2}s+∥\tilde{e}\left(t_{k+1}+T|t_{k+1}\right){∥}_{P}^{2}-{∥e^{*}\left(t_{k}+T|t_{k}\right)∥}_{P}^{2}. \end{array}$$

According to the feasible control input trajectory Eq. 10), we have $\tilde{u}\left(s|t_{k+1}\right)=u^{*}\left(s|t_{k}\right)$ and $\tilde{e}\left(s|t_{k+1}\right)=e^{*}\left(s|t_{k}\right)$ for $s\in [t_{k+1},\;t_{k}+T]$, then we have

$$\begin{array}{ccc} \Delta J_{i}^{*}\left(t_{k}\right) & \leq & \int_{t_{k}+T}^{t_{k+1}+T}∥\tilde{e}\left(s|t_{k+1}\right){∥}_{Q}^{2}+∥\tilde{u}\left(s|t_{k+1}\right){∥}_{R}^{2}ds+{∥\tilde{e}\left(t_{k+1}+T|t_{k+1}\right)∥}_{P}^{2}\\ & & -\;∥e^{*}\left(t_{k}+T|t_{k}\right){∥}_{P}^{2}-\int_{t_{k}}^{t_{k+1}}∥e^{*}\left(s|t_{k}\right){∥}_{Q}^{2}+{∥u^{*}\left(s|t_{k}\right)∥}_{R}^{2}s. \end{array}$$

As mentioned in feasibility analysis, $\tilde{e}\left(t_{k}+T|t_{k+1}\right)=e^{*}\left(t_{k}+T|t_{k}\right)$. For $s\in [t_{k}+T,\;t_{k+1}+T]$, the feedback control law $K\tilde{e}\left(s|t_{k+1}\right)$ is applied, then according to Lemma 1, we have $∥\tilde{e}\left(s|t_{k+1}\right){∥}_{Q}^{2}+∥\tilde{u}\left(s|t_{k+1}\right){∥}_{R}^{2}={∥e\left(t\right)∥}_{Q^{*}}^{2}\leq -\dot{V}\left(\tilde{e}\left(s|t_{k+1}\right)\right)$, and it can be obtained that

$$\begin{array}{ccc} & & \Delta J_{i}^{*}\left(t_{k}\right)\leq \int_{t_{k}+T}^{t_{k+1}+T}∥\tilde{e}\left(s|t_{k+1}\right){∥}_{Q}^{2}+∥\tilde{u}\left(s|t_{k+1}\right){∥}_{R}^{2}ds+{∥\tilde{e}\left(t_{k+1}+T|t_{k+1}\right)∥}_{P}^{2}\\ & & -\;∥e^{*}\left(t_{k}+T|t_{k}\right){∥}_{P}^{2}-\int_{t_{k}}^{t_{k+1}}∥e^{*}\left(s|t_{k}\right){∥}_{Q}^{2}+{∥u^{*}\left(s|t_{k}\right)∥}_{R}^{2}s\\ & & \leq -\int_{t_{k}}^{t_{k+1}}∥e^{*}\left(s|t_{k}\right){∥}_{Q}^{2}+{∥u^{*}\left(s|t_{k}\right)∥}_{R}^{2}s<0. \end{array}$$

Take $J^{*}\left(t_{k}\right)$ as the Lyapunov candidate, which is monotonically decreasing and indicate that the tracking error of mixed platoon system with the initial states $e\left(t_{0}\right)\in Z\setminus \Omega \left(\varepsilon \right)$ can enter the terminal set.

2) $e^{*}\left(s|t_{k}\right)\in \Omega \left(\varepsilon \right)$

When the tracking error enters the terminal set, according to Lemma 1, we have $\dot{V}\left(e(t)\right)\leq -{∥e\left(t\right)∥}_{Q^{*}}^{2}$ and e(t) converges to zero.

By combining the two parts, including outside the terminal set and in the terminal set, we have proved the closed-loop stability of the proposed MPC strategy in mixed platoon control.  □

Remark 9 In Theorem 2, the implied condition for ensuring closed-loop stability is the control period (update period) $t_{k+1}-t_{k}\geq \bar{\tau }$, and the prediction horizon satisfies $T\geq t_{k+1}-t_{k}\geq \bar{\tau }$. In the implementation, $\bar{\tau }$ can be obtained by field testing, and the other parameters can be selected according to Lemma 1.

Then we present the head-to-tail string stability analysis for the proposed MPC controller (7).

Theorem 3 *Suppose* Assumptions 1*–*2 *hold, then system (*5*) with the proposed MPC controller (7) satisfy head-to-tail string stability*.

Proof According to Theorem 1, the proposed MPC is feasible at all times, then we have $|\Delta v_{n}|\leq \Delta v_{max}$ at each time instant $t_{k}$ by the tracking error constraint Eq. 7f. Hence, for any external input $d_{0}$, the velocity fluctuations are suppressed with a bound from the head CAV to the tail CAV, but allowed to amplify between HDVs, which shows that the mixed platoon is head-to-tail string stable in the sense of $L_{2}$ string stability [20]. □

Remark 10 To the best of the authors’ knowledge, head-to-tail string stability of MPC-based mixed platoon control under communication delays has been seldom studied. To fill this gap, this paper incorporates constraint Eq. 7f into the proposed delay-compensating update mechanism to guarantee head-to-tail string stability.

## 6 Simulation validation

In this section, we build the platform in Matlab R2019b on a laptop equipped with Intel (R) Core (TM) i5–8300H CPU @2.30 GHz and 16GB RAM. The fmincon function is used to solve the optimization problem. The effectiveness of the proposed method is verified in the following aspects: robustness, fuel economy, and tracking performance.

### 6.1 Simulation setting

**Fig. 4:**
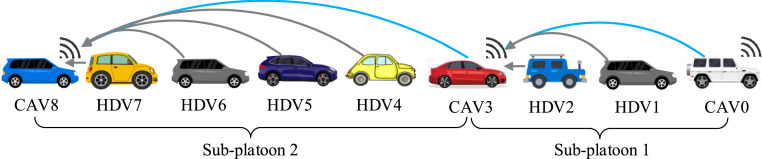
**The heterogeneous mixed platoons in the simulation**.

The heterogeneous mixed platoon includes 3 CAVs and 6 HDVs, as shown in Fig. 4. Time-varying heterogenous communication delays are imposed on all communication channels, and the delay bound is set as $\bar{\tau }=0.$2 s, which is above the typical bound for the end-to-end delay of the IEEE 802.11p vehicular networks [47]. The sampling interval is chosen as 0.1 s, the upper and lower bounds of the control input are set as $3\;m/s^{2}$ and $-5\;m/s^{2}$, the constant time lag of each vehicle is set as: $\delta_{0}=0.10,\delta_{1}=0.10,\delta_{2}=0.20,\delta_{3}=0.15,\delta_{4}=0.20,\delta_{5}=0.10,\delta_{6}=0.15,\delta_{7}=0.20,\delta_{8}=0.10$, the dynamical model for the HDVs is set as: $h_{s}=5$, $h_{g}=25,v_{max}=30$,$\;v^{*}=15$, $h^{*}=15m,\;\alpha_{1}=0.60$,$\alpha_{2}=0.62$,$\alpha_{4}=0.61$,$\alpha_{5}=0.63$,$\alpha_{6}=0.65$,$\alpha_{7}=0.63,\beta_{1}=0.90$,$\beta_{2}=0.85$,$\beta_{4}=0.87$,$\beta_{5}=0.83$,$\beta_{6}=0.90$,$\beta_{7}=0.84$.

1) *Comparison Design*

To show the effectiveness of the proposed controller, the following 2 comparisons are conducted:

• ***All HDVs***: In this case, the behavior of all vehicles follows the HDV model Eq. 2.
• ***Benchmark controller***: We introduce a benchmark controller as follows [25] for performance comparison
  $$\begin{array}{c} u_{p}\left(t\right)=\sum_{j=0}^{p-1}\alpha_{p,j}\left[g\left(h_{p,j}\left(t-\tau_{j}\left(t\right)\right)\right)-v_{p}\left(t-\tau_{j}\left(t\right)\right)\right]\\ +\sum_{j=0}^{p-1}\beta_{p,j}\left[v_{j}\left(t-\tau_{j}\left(t\right)\right)-v_{p}\left(t-\tau_{j}\left(t\right)\right)\right], \end{array}$$

where p is the index of CAV, $h_{p,j}$ is the relative spacing between HDV j and CAV p, $\alpha_{p,j},\beta_{p,j}$ are the response gains to the relative spacing and relative velocity. In the simulation, $\alpha_{p,j}=\alpha_{p},\beta_{p,j}=\beta_{p},p=\left\{3,8\right\}$, and other simulation settings are the same as above.

2) *Measurement of Effectiveness*

The adopted measurements of effectiveness (MOE) are the Fuel Consumption (**FC**) and Average Velocity Error (**AVE**) to reflect fuel economy and tracking performance of the controller. Here FC is calculated using the numerical model in [48], and AVE is defined as the average absolute error of velocity for all vehicles in the platoon.

3) *Controller Setting*

For the parameters of the MPC controller, weight matrices $Q_{i},\;R_{i}$ are selected by the desired control performance and can be tuned by trial and error. Based on the platoon model and weight matrices, feedback control gain $K_{i}$ is calculated with the linear quadratic regulator (LQR), then $P_{i}$ is calculated with the Lyapunov equations, after which $\varepsilon_{i}$ can be determined with the approach in [45,49]. The parameters are as follows: $u_{min}=-5$, $u_{max}=3$, $\Delta h_{min}=-6$, $\Delta h_{max}=6$, $\Delta v_{min}=-7$, $\Delta v_{max}=7$, $\Delta a_{min}=-3$, $\Delta a_{max}=3$, $T_{3}=0.2$, $T_{8}=0.3$, $\gamma_{3}=0.01$, $\gamma_{8}=0.01$, $\varepsilon_{3}=3.5$,$\varepsilon_{8}=5$, $Q_{3}=diag\left(0.5,0.5,0.1,0.5,0.5,0.1,0.5,0.5,0.1\right)$, $Q_{8}=diag\left(0.4,0.5,0.1,0.4,0.5,0.1,0.4,0.5,0.1,0.4,0.5,0.1,0.4,0.5,0.1\right)$, $R_{3}=0.1$, $R_{8}=0.3$ $K_{3}=\left[0.07,0.54,0.05,0.18,1.26,0.23,2.24,-3.58,-0.75\right]$, $K_{8}=\left[0.01,0.06,0.01,0.12,0.14,0.01,0.05,0.31,0.04,0.12,0.66,0.12,1.15,-2.18,-0.33\right]$.

### 6.2 Simulation results

Two case studies are conducted to show the performance of the proposed controller.

*1) Case 1: Emergency Braking*
Case 1 is a typical emergency case in real traffic flow and can verify the tracking performance and security of the algorithm [17]. Specifically, the desired trajectory of the leading CAV is given by
  $$v_{0}=\left\{ \begin{array}{c} 15\;m/s,\;t<2s,\\ 15-5t\;m/s,\;2s\leq t<4s,\\ 5\;m/s,\;4s\leq t<9s,\\ 5+2t\;m/s,\;9s\leq t<14s,\\ 15m/s,\;t\geq 14s. \end{array}\right.$$

**Fig. 5:**
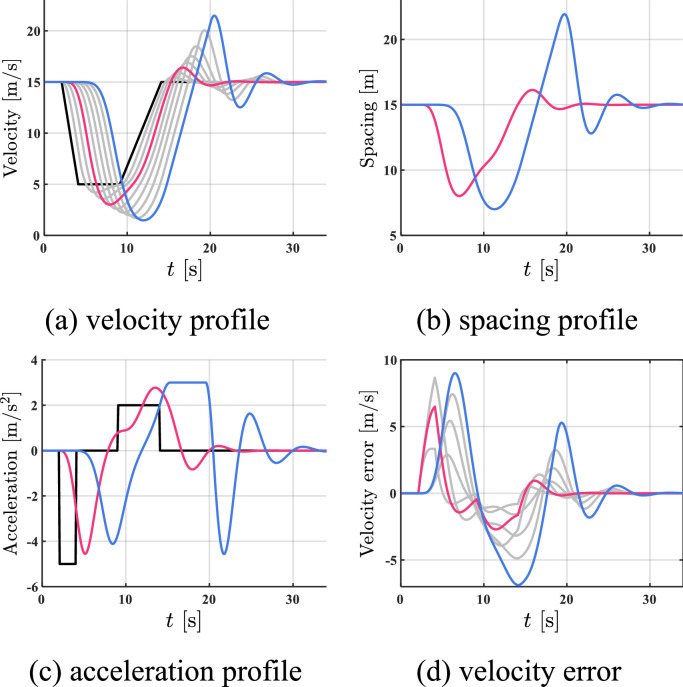
**Profiles of states when all the vehicles are HDVs**.

**Fig. 6:**
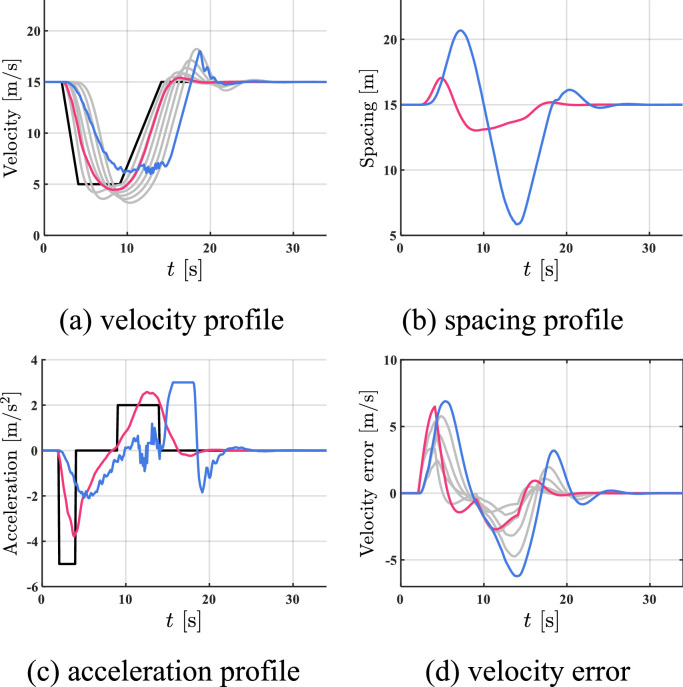
**Profiles of states with two CAVs utilizing the benchmark controller**.

**Fig. 7:**
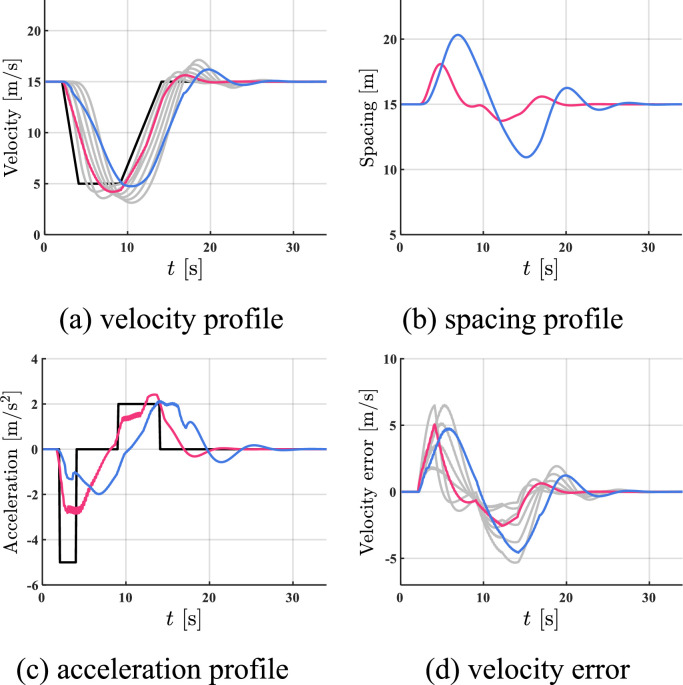
**Profiles of states with two CAVs utilizing the proposed controller**.

The simulation results of all HDVs, the benchmark controller, and the proposed controller are shown in Fig. 5, Fig. 6–Fig. 7. The red profile and the blue profile represent CAV 3 and CAV 8, respectively. In spacing profiles and acceleration profiles, the profiles of HDVs are hidden (Table 1).

**Table 1:** **Comparison results in case 1**.

	All HDVs	Benchmark controller	Proposed controller
FC [mL]	577.13	501.10	472.06
AVE [m/s]	2.02	1.39	0.93

As shown in Fig. 5–Fig. 7, the velocity fluctuations magnify along the platoon with fully HDV, while both the benchmark controller and the proposed controller can smooth traffic flow with 2 following CAVs. Compared with the proposed controller, the state profiles utilizing the proposed controller are smoother and the tracking errors are much smaller. In addition, the velocity error can be constrained in a safe range by the proposed controller, which indicates head-to-tail string stability in Theorem 3. For quantitative comparison, a 5.8% reduction in FC and a 33.1% reduction in AVE can be observed after introducing the proposed controller compared to the case of the benchmark controller in Table I, while more improvement is exhibited compared to the case of all HDVs. The proposed controller can lead to better performance in robustness, fuel economy, and tracking.

The average computation time of the proposed controller is 0.18 s, which is acceptable since the computation period is 0.2 s under the proposed control scheme. The computational burden can be greatly reduced by the proposed synchronization mechanism and dual-mode scheme, since the synchronization mechanism can reduce the computation frequency, and the feedback controller in mode 2 is time-efficient. In practical applications, faster solvers like IPOPT can be deployed on higher computational power devices to improve real-time performance [34].

2) Case 2: Sinusoidal Perturbation
In Case 2, a sinusoidal perturbation is imposed on the leading CAV to verify the tracking performance in dynamic tracking scenarios.

**Fig. 8:**
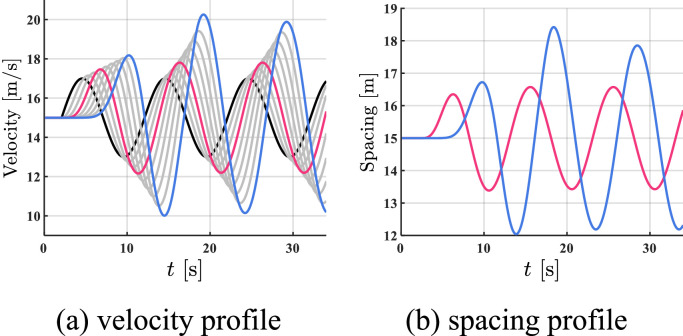
**Profiles of states when all the vehicles are HDVs**.

**Fig. 9:**
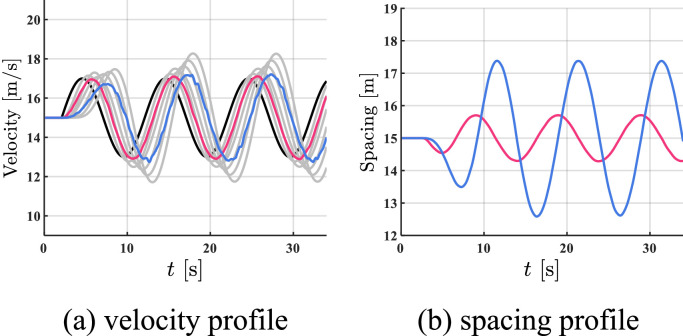
**Profiles of states with two CAVs utilizing the benchmark controller**.

**Fig. 10:**
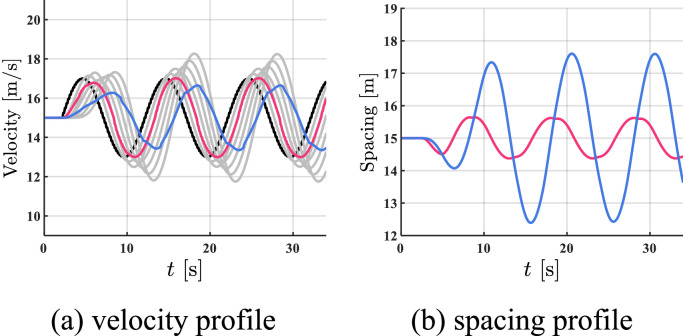
**Profiles of states with two CAVs utilizing the proposed controller**.

The simulation results of all HDVs, the benchmark controller, and the proposed controller are shown in Fig. 8, Fig. 9–Fig. 10. The red profile and the blue profile represent CAV 3 and CAV 8, respectively. In spacing profiles, the profiles of HDVs are hidden (Table 2).

**Table 2:** **Comparison results in case 2**.

	All HDVs	Benchmark controller	Proposed controller
FC [mL]	713.98	581.30	555.37
AVE [m/s]	2.16	1.46	1.05

As shown in Fig. 8–Fig. 10, in the dynamic tracking scenario, the velocity and spacing fluctuations are smaller utilizing the proposed controller than in the case of fully HDV and utilizing the benchmark controller, which confirms contribution Eq. 3. As shown in Table II, by utilizing the proposed controller, a 4.5% reduction in FC and a 28.1% reduction in AVE can be observed compared to the benchmark controller, which further verifies the effectiveness of our strategy.

### 6.3 Validation in additional mixed traffic flow

**Fig. 11:**
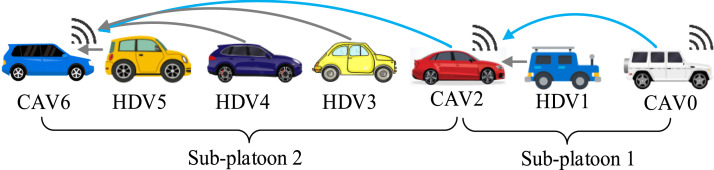
**Additional heterogeneous mixed platoons in the simulation**.

To further verify the effectiveness of the proposed method in different traffic flow, another heterogenous mixed platoon including 3 CAVs and 4 HDVs in Fig. 11 is established. The dynamical model for the HDVs is set as: $h_{g}=35$,$\;v^{*}=15$, $h^{*}=20m,\;\alpha_{1}=0.60$,$\alpha_{3}=0.65$,$\alpha_{4}=0.61$,$\alpha_{5}=0.63$,$\beta_{1}=0.90$,$\beta_{3}=0.80$,$\beta_{4}=0.87$,$\beta_{5}=0.83$, and the other settings are the same as above.

**Fig. 12:**
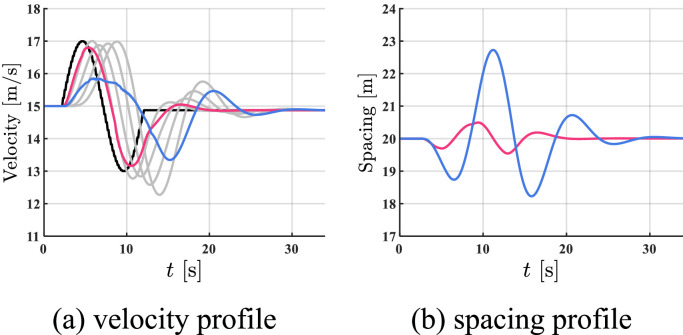
**Profiles of states in additional traffic flow utilizing the proposed controller**.

In this case, a sinusoidal perturbation is imposed on the leading CAV from t=2s to t=12s, and the results are shown in Fig. 12. The red profile and the blue profile represent CAV 2 and CAV 6, respectively. In spacing profile, the profiles of HDVs are hidden.

As shown in Fig. 12, the proposed controller can smooth traffic flow and guarantee stability under communication delays in the additional scenario. This indicates the effectiveness of the proposed method in different traffic flows.

## 7 Conclusion

This paper has proposed an MPC-based method for the cooperation of heterogeneous mixed platoons subject to time-varying communication delays. In the MPC scheme, a serial HDV state prediction mechanism and a delay-compensating update mechanism have been designed to compensate for communication time delays. The robustness constraint and tracking error constraint have been formulated to guarantee feasibility, closed-loop stability, and head-to-tail string stability. Numerical results have revealed that the proposed method outperformed the state-of-the-art consensus-based controller in stability, fuel economy, and dynamic tracking.

In future work, one topic is to consider the prediction uncertainty of HDVs. Besides, problems such as driver reaction time, unknown HDV parameters, and online computation also merit further study to improve real-world implementation.

## Declaration of competing interests

The authors declare that they have no conflicts of interest in this work.
